# Comparative transcriptomic provides novel insights into the soybean response to *Colletotrichum truncatum* infection

**DOI:** 10.3389/fpls.2022.1046418

**Published:** 2022-11-25

**Authors:** Thaís R. Boufleur, Nelson S. Massola Júnior, Sioly Becerra, Elena Baraldi, Líllian B. J. Bibiano, Serenella A. Sukno, Michael R. Thon, Riccardo Baroncelli

**Affiliations:** ^1^ Department of Plant Pathology and Nematology, Luiz de Queiroz College of Agriculture (ESALQ), University of São Paulo (USP), Piracicaba, Brazil; ^2^ Department of Microbiology and Genetics, Institute for Agribiotechnology Research (CIALE), University of Salamanca (USAL), Villamayor, Spain; ^3^ Department of Agricultural and Food Sciences (DISTAL), University of Bologna, Bologna, Italy

**Keywords:** time-course, RNA sequencing, gene expression, *Glycine max*, anthracnose

## Abstract

**Introduction:**

Soybean (*Glycine max*) is among the most important crops in the world, and its production can be threatened by biotic diseases, such as anthracnose. Soybean anthracnose is a seed-borne disease mainly caused by the hemibiotrophic fungus *Colletotrichum truncatum*. Typical symptoms are pre- and post-emergence damping off and necrotic lesions on cotyledons, petioles, leaves, and pods. Anthracnose symptoms can appear early in the field, causing major losses to soybean production.

**Material and Methods:**

In preliminary experiments, we observed that the same soybean cultivar can have a range of susceptibility towards different strains of *C. truncatum*, while the same *C. truncatum* strain can cause varying levels of disease severity in different soybean cultivars. To gain a better understanding of the molecular mechanisms regulating the early response of different soybean cultivars to different *C. truncatum* strains, we performed pathogenicity assays to select two soybean cultivars with significantly different susceptibility to two different *C. truncatum* strains and analyzed their transcriptome profiles at different time points of interaction (0, 12, 48, and 120 h post-inoculation, hpi).

**Results and Discussion:**

The pathogenicity assays showed that the soybean cultivar *Gm*1 is more resistant to *C. truncatum* strain 1080, and it is highly susceptible to strain 1059, while cultivar *Gm*2 shows the opposite behavior. However, if only trivial anthracnose symptoms appeared in the more resistant phenotype (MRP; *Gm*1-1080; *Gm*2-1059) upon 120 hpi, in the more susceptible phenotype (MSP; *Gm*-1059; *Gm*2- 1080) plants show mild symptoms already at 72 hpi, after which the disease evolved rapidly to severe necrosis and plant death. Interestingly, several genes related to different cellular responses of the plant immune system (pathogen recognition, signaling events, transcriptional reprogramming, and defense-related genes) were commonly modulated at the same time points only in both MRP. The list of differentially expressed genes (DEGs) specific to the more resistant combinations and related to different cellular responses of the plant immune system may shed light on the important host defense pathways against soybean anthracnose.

## 1 Introduction

Soybean (*Glycine max*) is among the top-produced crops in the world (Hartman et al., 2011) and can be affected by several diseases, such as anthracnose caused by *Colletotrichum* spp. (Sharma et al., 2011; Yang and Hartman, 2015a; Boufleur et al., 2021a), which can lead to up to 90 kg/ha of losses for each percent unit of disease incidence in the field (Dias et al., 2016). A survey based on publicly available genetic data revealed that at least 12 *Colletotrichum* lineages associated with soybean, with species of the *C. truncatum* and *C. orchidearum* species complexes (s.c.) the most prominent worldwide. Among these, members of the *C. truncatum* s.c. are the most frequently identified from symptomatic soybean plants (Boufleur et al., 2021a). The *C. orchidearum* s.c. includes three species, namely, *C. musicola*, *C. plurivorum*, and *C. sojae*, which have recently been described as infecting soybean (Barbieri et al., 2017; Rogério et al., 2017; Boufleur et al., 2020; Boufleur et al., 2021a). Although there are pathogenic races for other *Colletotrichum* species (Gela et al., 2021; Nunes et al., 2021), no races are reported for *C. truncatum* associated with soybean or other hosts (Rao and Nandineni, 2017; Rogério et al., 2017; Dias et al., 2019)

*C. truncatum* has a hemibiotrophic lifestyle (Latunde-Dada and Lucas, 2007), and typical anthracnose symptoms can appear in all plant tissues during all the physiological stages of soybean, depending on the environmental conditions. In newly cultivated areas, the main source of inoculum of *C. truncatum* are infected seeds. Therefore, the first symptoms can appear in the early stages of soybean planting in the field, leading to pre- and post-emergence damping off and stand losses (Boufleur et al., 2021a). The control of the disease relies on the use of *Colletotrichum* spp.-free seeds, cultural practices, and fungicides. Although chemical control is an effective strategy for disease management and has helped farmers to enhance productivity due to reduced pest and disease losses, reports of *C. truncatum* resistance against the most common fungicides are increasing over the years (Torres-Calzada et al., 2015; Poti et al., 2020; Rogério et al., 2022). Genetic resistance is a more environment- friendly solution for efficient production systems (Nelson et al., 2018), and soybean breeding against anthracnose should be employed as part of a strategy to control this disease.

The plant immune system is complex, continuous, and capable of evolving over time to detect pathogen and microbe invasions (Cook et al., 2015). Overall, the system accounts for two tiers, namely, pattern-triggered immunity (PTI) and effector-triggered immunity (ETI) (Jones and Dangl, 2006), sharing many signaling components that cross-communicate and interact to raise harmonized immune responses against any biotic challenge (Cook et al., 2015; Yu et al., 2017; Kanyuka and Rudd, 2019). Next-generation sequencing (NGS) technologies such as RNA sequencing (RNAseq) can be useful tools to approach the complex molecular networks acting in plant immunity. For example, transcriptional profiling has provided a great contribution to elucidating key mechanisms differently involved in plant defense pathways against *Colletotrichum* species (O’Connell et al., 2012; Bhadauria et al., 2015; Padder et al., 2016; Bhadauria et al., 2017; Zhu et al., 2022). A recent study investigated the expression profiles of detached soybean pods of a soybean wild- type (wt) and mutant genotype during infection with *C. truncatum* and revealed that a higher level of resistance of the mutant (derived from γ-rays irradiated ZC3 seeds) was associated with the overexpression of genes associated with signaling pathways control, transcription reprogramming, R-gene, and PR-protein activation (Zhu et al., 2022). However, these transcriptome studies have focused on the reproductive stages of one single soybean cultivar in its wt and mutant form and only one strain of the pathogen.

In this study, we present a broad transcriptome investigation of the soybean –*C. truncatum* interaction during the early phenological stages of soybean. In particular, we sequenced the transcriptomes of two commercial soybean cultivars that alter their response upon infection with two strains of *C. truncatum* over time, including the early, middle, and late stages of the disease. We investigated the differences in the plant response between a more resistant and a more susceptible phenotype. The combined approach revealed a strong correlation between susceptibility and transcription profiles of major genes involved in the response of soybean toward *C. truncatum*.

## 2 Materials and methods

### 2.1 Plant materials and inoculation

The differences in the response of soybean during *C. truncatum* infection were assessed in four combinations of two commercial soybean cultivars from Monsoy IPRO7739 (*Gm1*) and IPRO8372 (*Gm2*) and two *C. truncatum* strains CMES1059 (1059) and CMES1080 (1080). To perform the pathogenicity assays, disinfected soybean seeds [NaClO 1% for 1 min, followed by three times in sterile distilled water (SDW)] were placed in Petri dishes (90 × 90 mm) filled with 100 g of sterile sand, soaked with 10 ml of SDW and incubated for 32 h at 25°C until germination. Conidial suspensions of *C. truncatum* were harvested from 15-day-old cultures and adjusted to a final concentration of 1 × 10^6^ conidia/ml. Three seedlings for each treatment were inoculated as described by Dubrulle et al. (2020), and SDW was used as a negative control. Inoculated seedlings were incubated in the dark, at 25°C for 4 h and then transferred to 50- ml pots filled with sterilized vermiculite. Pots were randomly distributed in a Conviron plant growth chamber at 25°C, with 12 h of photophase, until symptoms appeared. Three biological repetitions were performed.

To estimate the severity of anthracnose disease for each combination (1059 -*Gm1*, 1080 -*Gm1*, 1059-*Gm2*, and 1080-*Gm2*), symptoms were evaluated at 120 hpi using a diagrammatic scale that ranges from 0 to 5 adapted from Yang and Hartman (2015b) (Boufleur et al., 2021b). Three biological replicates were performed. Disease severity was compared with a double factorial scheme analysis using the ExpDes R package v.1.2.0 (Ferreira et al., 2014) with the *post-hoc* Tukey method at a 0.05 significance level. Based on the similarity of the defense response, the treatments were arranged into two groups: “MRP” as the more resistant phenotypes (*Gm*1-1080; *Gm*2-1059) and “MSP” as the more susceptible phenotypes (*Gm*-1059; *Gm*2- 1080). The confirmation of the presence of *C. truncatum* in inoculated plants was performed by reisolating the fungus 120 h post-inoculation (hpi) and by species-specific polymerase chain reaction (PCR) of the *glyceraldehyde-3-phosphate dehydrogenase* (GAPDH) gene, using ColTF6 and R5 primers as described by (Ciampi-Guillardi et al., 2020).

### 2.2 Total RNA isolation, library construction, and RNA sequencing

To perform total RNA extraction, soybean plantlets were inoculated as described in *Section 2.1*. Hypocotyl fragments (0.5 cm) of five independent soybean plants were grouped as a single experimental replicate, and three biological replicates were analyzed for each treatment at 0, 12, 48, and 120 hpi. To avoid degradation, the harvested plant tissues were frozen immediately in liquid nitrogen and stored at −80°C. Total RNA was purified using the PureLink RNA Mini Kit (Invitrogen, USA) following the manufacturer’s instructions and treated with DNAse (Life Technologies, USA) to remove DNA contamination. A Qubit 2.0 fluorometer (Life Technologies, USA) was used to estimate the amount of total RNA extracted, and the integrity was checked using Agilent TapeStation 4200 (Agilent Technologies, USA).

A total of 48 libraries were prepared using the NEBNext Ultra RNA Library Prep Kit for Illumina (NEB, USA) following the manufacturer’s instructions. Sequencing libraries were validated on Agilent TapeStation (Agilent Technologies, USA) and quantified by quantitative PCR (KAPA Biosystems, South Africa) and by Qubit 2.0 fluorometer (Invitrogen, USA). Libraries were prepared and sequenced at Genewiz (South Plainfield, USA) using Illumina HiSeq4000 (2 × 150 bp).

### 2.3 Quantification of transcript abundance and time-course differential expression analyses

The quality of reads was assessed using FastQC v.0.11.7 (Andrews, 2010), and sequence adapters were filtered using CutAdaptors v.1.9.1 (Martin, 2011). Paired-end clean reads were mapped to the *G. max* reference genome (Wm.82.a2.v1) (Schmutz et al., 2010) using HISAT v.2.1.0 (Kim et al., 2015). Expression values of transcripts for each library were estimated with StringTIE v.1.3.5 (Pertea et al., 2016).

A principal component analysis (PCA) was performed to check the consistency of the replicates, using the plotPCA function of DESeq2 v. 1.28.1 (Love et al., 2014). The expression profiles of soybean transcripts in MRP and MSP were accessed with TCseq R package v.1.12.1 using the *k-means* method (Wu and Gu, 2022). The differentially expressed genes (DEGs) were identified based on a time-course approach using DESeq2 v.1.28.1 (Love et al., 2014). Transcripts with ≤10 counts per million in the three repetitions were excluded from the analysis. The DEGs were filtered based on a false-discovery rate (FDR) of ≤ 0.05 and log2 fold change (log2FC), and only ≥ 2 or ≤ −2 was considered DE.

Venn diagrams of DEGs were constructed to identify transcripts related to the MRP and the MSP at each time point using the Venn Diagram module of Intervene online tools (Khan and Mathelier, 2017). Selected transcripts were grouped into functional categories accordingly to annotations of the soybean reference genome and manually checked using InterProScan and PFAM terms. Overrepresented biological processes (BPs) and molecular functions (MFs) among common up- and down regulated transcripts in each condition were predicted with the BiNGO app in the Cystoskape v.3.8.1 (Shannon et al., 2003; Maere et al., 2005), using the hypergeometric exact tests with an FDR ≤ 0.05.

## 3 Results

### 3.1 Inversion of the physiological response of soybean cultivars upon inoculation with different strains of *C. truncatum*

**Figure 1:**
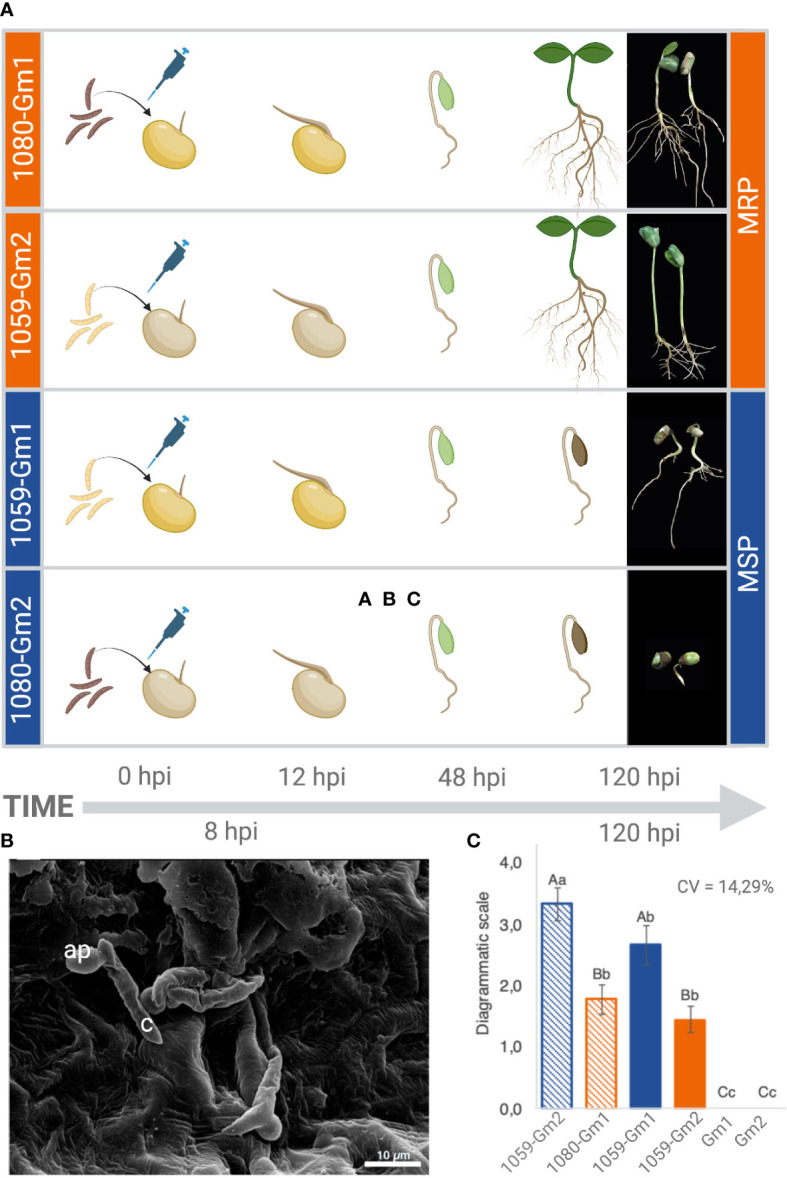
Physiological response of two soybean cultivars (*Gm1* and *Gm2*) upon inoculation with two *Colletotrichum truncatum* strains (1080 and 1059). **(A)** Scheme of the evolution of anthracnose symptoms observed at 0, 12, 48, and 120 h post-inoculation (hpi). **(B)** Representative scanning electron micrograph of *C. truncatum* conidia germinating on soybean seeds 8 hpi. ap, appressoria; c, conidia. **(C)** Biological groups formed after evaluation of the level of disease in the different combinations based on results of the Tukey test (≤0.05) applied to transformed data ((X+1)ˆ0.05). Upper case equal letters do not differ in the average strain level inside each cultivar; lower case equal letters do not differ in the average of cultivar level inside each strain. More resistant phenotypes (MRPs) are represented in orange, while more susceptible phenotypes (MSPs) are represented in blue. Created with BioRender.

To determine the physiological response of two soybean cultivars upon inoculation with *C. truncatum*, seedlings were inoculated as described by Dubrulle et al. (2020). Preliminary studies showed that *C. truncatum* conidia germinate and form appressoria within 6 h post-inoculation (hpi) on the surface of soybean seedlings (**Figure 1**). Initial anthracnose symptoms appeared at 72 hpi and evolved to severe necrosis by 120 hpi in the 1080-*Gm*2 and 1059-*Gm1* strains–soybean cultivars (**Figure 1A**), while at the same time point, symptoms in 1080-*Gm1* and 1059-*Gm2* appeared to be milder. Plants used as negative controls remained asymptomatic.

The evaluation of the severity of the symptoms at 120 hpi resulted in two biological groups, based on the level of susceptibility of each soybean cultivar to the different fungal strains. A higher level of resistance was observed in *Gm1* interacting with 1080 strain and *Gm2* interacting with 1059 (MRP), while a higher level of susceptibility was observed for *Gm2* interacting with 1080 and *Gm1* interacting with 1059 (MSP) (**Figure 1**). Both 1080 and 1059 strains were successfully reisolated from all the inoculated combinations, and the presence of *C. truncatum* was confirmed in soybean plants during the symptomatic and asymptomatic phases of the disease using species-specific PCR (**Supplementary Figure S1**).

### 3.2 The physiological responses observed in soybean cultivars upon infection with *C. truncatum* were confirmed by transcriptomic profiles in both phenotypes

The transcriptomic reprogramming of soybean during the development of *C. truncatum* infection was analyzed based on RNA sequencing at 0, 12, 48, and 120 hpi, with three biological replicates for each treatment at each time point. A total of 1,202,534,283 reads were obtained for the 48 libraries constructed. The clean reads were aligned to the soybean reference genome with an average mapping percentage of 89%. The PCA of normalized samples showed that the three independent biological replicates generated for each treatment are highly correlated, demonstrating the robustness of the experimental protocol and the analysis pipeline (**Supplementary Figure 2**; **Supplementary Table S1**).

**Figure 2:**
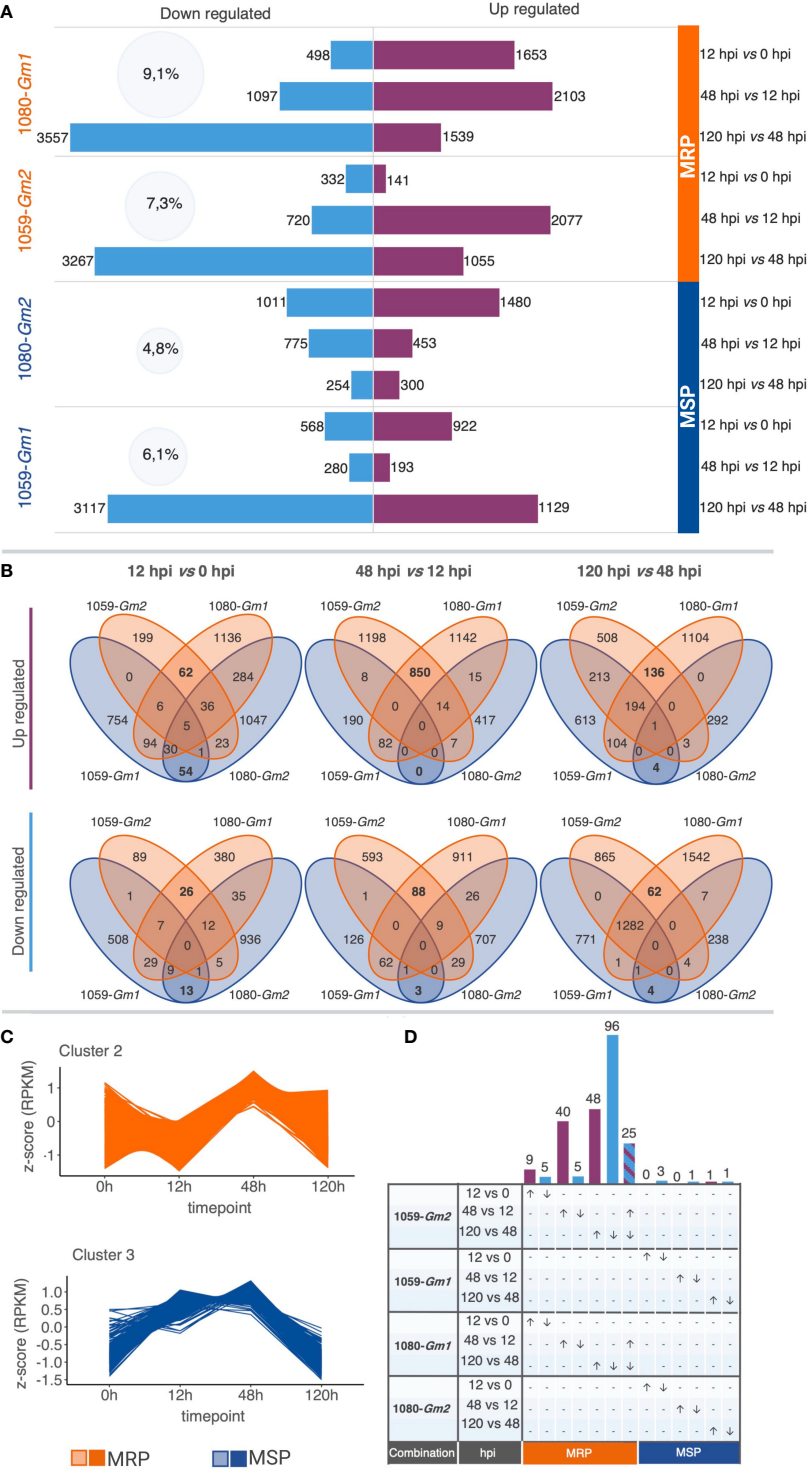
Overview of the soybean transcriptomic reprogramming upon infection with different strains of *Colletotrichum truncatum* in more resistant phenotype (MRP: 1080-*Gm1*, and 1059-*Gm2*) and more susceptible phenotype (MSP: 1059-*Gm1* and 1080-*Gm2*). **(A)** Total changes in soybean transcriptome. Numbers inside the light blue cycles indicate the percentage of changes in the soybean transcriptome for each combination. The number of differentially expressed transcripts (DEGs) in each time pairwise comparison is represented by purple (up regulated) and light blue (down regulated) bars. **(B)** Venn diagrams displaying the overlaps of DEGs among combinations of MRP and MSP, at different time pairwise comparisons. **(C)** Representative *k-*means clusters corresponding to the majority of DEGs identified as common at MRP (cluster 2) or MSP (cluster 3). **(D)** UpsetR plot showing differentially expressed genes specific to each time pairwise comparison for MRP and MSP. The number of up regulated (purple bars, arrows directed upwards) and down regulated (light blue bars, arrow directed downward) genes are represented on the upper side of each intersection bar. Genes that are up regulated at 48 h post-inoculation (hpi) and down regulated at 120 hpi are colored with purple and blue diagonal stripes. Created with BioRender.

Gene expression patterns were determined for both interactions using the *k-means* method to cluster raw transcript counts. Results showed a higher pattern of temporal synchronization in the MRP (10,222 genes in clusters) when compared to the MSP (3,004 genes in clusters). Forty-eight percent of the transcripts in MRP show a peak of expression at 48 hpi, while 33% of the transcripts of MSP show a peak of expression at 12 hpi. On the other hand, the MSP have a higher number of transcripts (26%) with a peak of expression starting at 12 hpi and maintained until 48 hpi when compared with the MRP (15%) (**Figure 2**; **Supplementary Figure S2**).

### 3.3 A higher coordinated transcriptomic reprogramming was observed in MRP as compared to the MSP

A total of 13,478 DEGs were identified in the 12 time-course comparisons (**Figure 2**; **Supplementary Table S2**). Among these, 5,495 were DE only in the MRP, 2,979 were DE only in the MSP, and 4,904 were DE in both. The highest percentage of changes in the soybean transcriptome was observed for the MRP when compared to the MSP. In both interactions of the MRP, up- and down-regulated genes followed the same quantitative pattern of expression, and the number of DEG increases proportionally with time, while in the MSP, each interaction has a unique pattern of expression. In the MSP combination 1080-*Gm2*, a decrease in the total DEG can be observed in time, while in the other MSP 1059-*Gm1*, a decrease in DEGs can be observed between 12 and 48 hpi, whereas an increase in DEGs becomes apparent between 48 and 120 hpi (**Figure 2**).

To identify patterns associated with soybean resistance to anthracnose, regardless of soybean cultivar and *C. truncatum* strain, the relationships among all treatments were visualized (**Figures 2B, D**). Overall, the MRP shares the highest number of DEGs at each time point when compared to the MSP with the maximum of up regulated (850) and down regulated (88) genes observed at 48 vs. 12 hpi. A total of 1,512 overlapping genes of MRP or MSP on at least one timing were selected for downstream analysis. Of those, 1,401 have a synchronized expression only in the MRP, of which 431 are not modulated in the MSP; 31 had a synchronized expression only in the MSP, and among these, 12 are not modulated in the MRP. The pattern of expression of 72% of the selected genes of the MRP and 25% of the selected genes of the MSP is represented by *k-*means clusters (**Figure 2**).

We performed bioinformatic analysis to identify genes involved in different stages of soybean defense against *C. truncatum*. A total of 235 genes involved in several defense responses were DE in specific timings and selected for further analysis. From those, 229 genes are specific to the MRP, while only 6 genes were identified as specific to the MSP. Except for 25 genes that were up regulated between 12 and 48 hpi and down regulated at 120 hpi, all the identified genes were associated with a specific timing (**Figure 2**). We hypothesize that genes modulated only in the MRP and related to specific time points would probably reflect the regulatory mechanisms conferring the more resistant response observed in the biological experiments. A list of the selected DEGs and their annotations is available in **Supplementary Table S3**.

### 3.4. Plant defense-related pathways are enriched in the MRP

A GO enrichment analysis was performed with the subset of genes reported in *Section 3.3* by scanning up- and down regulated DEG separately to identify overrepresented biological processes (BPs) and molecular functions (MFs) activated in soybean during interaction with *C. truncatum*. While no enriched GO terms were revealed for the MSP, for the MRP, the hypergeometric test (*p* ≤ 0.05) detected 42 BP and 40 MF up regulated between 48 and 12 hpi, 2 BP and 12 MF up regulated between 48 and 120 hpi, and 39 BP and 46 MF down regulated at the same timing. Among these, 32 enriched GO terms up regulated between 12 and 48 hpi are down regulated at 120 hpi (**Supplementary Figures S3**, **4**). These results indicate that most of the enriched processes are triggered in the early and middle stages of the development of soybean anthracnose and deactivated during the late stages. Several genes involved in plant defense mechanisms against biotic stresses are enriched, including MFs involved in pathogen molecular patterns recognition and binding, reactive oxygen species (ROS) and transcriptional reprogramming, and BPs involved in defense responses to biotic and abiotic stimuli (**Supplementary Figures S3**, **4**).

### 3.5 *C. truncatum* is recognized by soybean extracellular and intracellular receptors

**Figure 3:**
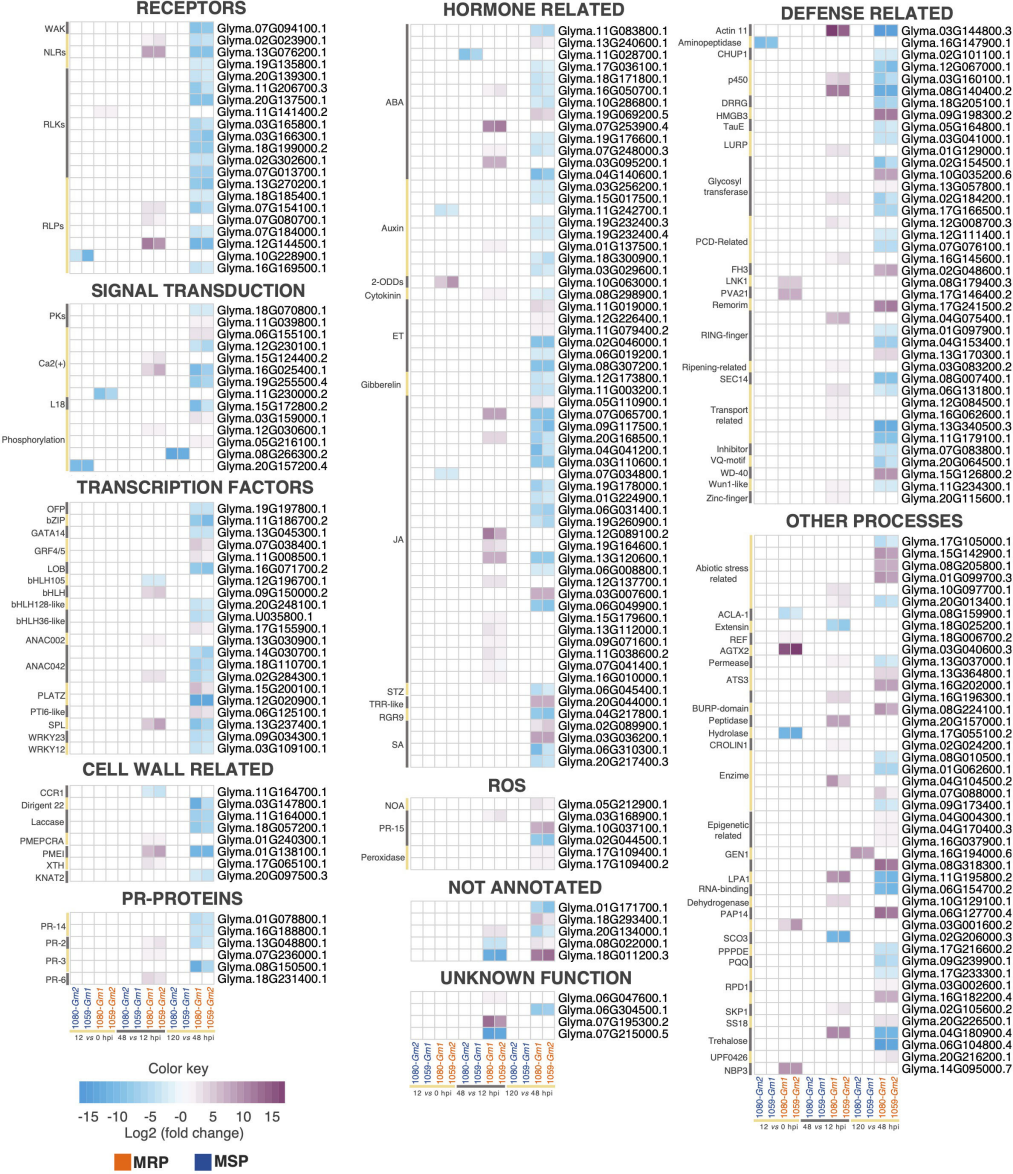
Heatmaps of differentially expressed (DE) transcripts related to specific time points after soybean inoculation with *Colletotrichum truncatum*. The annotated genes are grouped in different functional categories. The detailed annotation of the genes is provided at **Supplementary Table S3**. More resistant phenotype (MRP): 1080-*Gm1* and 1059-*Gm2*; more susceptible phenotype (MSP): 1059-*Gm1* and 1080-*Gm2*, hours post-inoculation (hpi).

Host plants can recognize pathogen-associated molecular patterns (PAMPs) or pathogen effectors due to their pattern-recognition receptors (PRRs) and/or nucleotide-binding leucine-rich repeat receptors (NLRs) (Jones and Dangl, 2006; Kanyuka and Rudd, 2019). Our results revealed the modulation of 51 PRRs and NLRs that are DE only in the MRP, of which 28 are receptor like-kinases (RLKs), 10 are receptor-like proteins (RLPs), 3 are wall-associated kinases (WAKs), 9 NLRs have a toll/interleukin-1 sensor, and 2 have a coiled-col (cc) sensor (**Figure 3**; **Supplementary Table S3**). The time-specific extracellular receptors included one leucine-rich-repeat (LRR) kinase up regulated between 0 and 12 hpi; one lectin (Lec)-RLP up regulated between 12 and 48 hpi; two Lec-RLPs up regulated between 12 and 48 hpi and down regulated between 48 and 120 hpi; and five LRR-RLKs, three GNK2, one wall-associated kinase (WAK), three Lec-RLPs, and one LRR-RLP down regulated between 48 and 120 hpi (**Figure 3**). Intracellular time-specific receptors included two TIR-NBS-LRRs up regulated between 12 and 48 hpi and down regulated at 120 hpi, and one CC-NBS-LRR down regulated between 48 and 120 hpi. Except for two LRR-RLKs (*Glyma.03G165800.1* and *Glyma.03G166300.1*) and a Lec-RLP (*Glyma.07g14100.1*), log2FC values were similar among both combinations. On the other hand, only one PRR was modulated only in the MSP, a Lectin (Lec)-RLK, down regulated within the first 12 hpi (**Figure 3**).

### 3.6 Signaling transduction mechanisms are activated upon *C. truncatum* recognition

#### 3.6.1 Reactive oxygen species and Ca^2+^ signaling pathways

The recognition of pathogen PAMPs or effectors leads to the activation of downstream signaling mechanisms such as changes in the cytosolic Ca^2+^ concentrations and ROS, which can interact to lead to a stronger immune response (Marcec et al., 2019). The respiratory burst oxidase homolog (RBOH) is one of the primary enzymes for ROS production upon pathogen infection and plays a central role in the interaction among Ca^2+^ and ROS signaling pathways (Marcec et al., 2019). In our work, an RBOH was up regulated between 12 and 48 hpi only in the MRP, and 13 genes related to the Ca^2+^ signaling pathway and 19 related to ROS were modulated in the same condition (**Figure 3**).

Five genes associated with Ca^2+^ signaling pathway are involved in the formation of the Ca^2+^ signal (encoders) (Marcec et al., 2019). From these, two are time specific: a transmembrane protein 64-like (TMEM64), up regulated between 12 and 48 hpi, and a cyclic nucleotide gated ion channel 1-like (CNGC) up regulated between 12 and 48 hpi and down regulated at 120 hpi. The Ca^2+^ sensors included 15 genes, 2 of which were time specific, including *Glyma.06G155100.1*, which was up regulated at 120 hpi, and *Glyma.12G230100.1*, which was down regulated at the same time. Finally, one Ca^2+^ decoder (WRKY28 transcription factor) was commonly up regulated for both combinations of the MRP between 12 and 48 hpi. Ten genes related to the production of ROS signals were identified, including five that were time specific: two RmlC-like cupins, one up regulated between 12 and 48 hpi and another up regulated at 120 hpi; and two peroxidases up regulated at 120 hpi and a pathogenesis- related (PR)-15 protein down regulated at 120 hpi. Another eight genes, related to the regulation of ROS, were modulated, including a time-specific P-loop containing nucleoside triphosphate hydrolase (NOA1) gene up regulated at 120 hpi.

#### 3.6.2 Plant hormone signaling

Plant hormones are central regulators of plant immunity, acting downstream of pathogen recognition and regulating signal transduction and plant defense activation (Berens et al., 2017; Aerts et al., 2021).

In our work, 90 genes involved in the biosynthesis of seven classes of hormones were commonly modulated and specific to the MRP or the MSP; from those, 59 were modulated at specific time points, including 23 related to jasmonic acid (JA), 9 to auxin (IAA), 12 to abscisic acid (ABA), 3 to salicylic acid (SA), 6 to ethylene (ET), 2 to gibberellin (GA), 1 to cytokinin, and 4 related to diverse hormone signaling pathways (**Figure 3**). These findings indicate that JA, IAA, and ABA pathways responded more strongly to *C. truncatum* infection in the MRP than SA, ET, and GA.

The only time-specific modulated gene in the MSP is an *armadillo repeat kinesin 3* (ARK3), which acts as an upstream signaler from IAA (Yee and Goring, 2009), and is down regulated between 48 and 12 hpi. On the other hand, all the other time-specific modulated genes are specific to the MRP. For JA, a lipoxygenase (LOXA) was down regulated at 12 hpi and a Wuschel-related homeobox 11 (WOX11) transcription factor up regulated at 120 hpi. All of the other time-specific genes involved in this pathway had a peak of expression at 48 hpi. Three genes involved in ET biosynthesis were overexpressed at 120 hpi, while four were repressed at the same time, indicating that they had a peak of expression at 48 hpi. For SA, only three genes were time specific, including two MATE efflux genes, involved in SA transport (Zhang and Li, 2019) up regulated at 120 hpi and a *yellow stripe-like 1* (YSL1) genes, which had a peak of expression at 48 hpi. For IAA, a *mizu-kussei 1-like* (MIZ1) was down regulated at 12 hpi, and all of the other genes had a peak of expression at 48 hpi. Two genes related to GA had a peak of expression at 48 hpi. A galactose oxidase gene related to the negative regulation of cytokinin was up regulated between 12 and 48 hpi and down regulated at 120 hpi. A 2-oxoglutarate/Fe(II)-dependent dioxygenase (DIOX2), involved in diverse hormone and secondary metabolite biosynthesis and catabolism pathways (Farrow and Facchini, 2014), was up regulated early at 12 hpi. A *salt tolerance zinc finger* (STZ) involved in JA crosstalk with other hormones (Zander et al., 2020) was down regulated between 48 and 120 hpi. A tetratricopeptide repeat (TPR)-like superfamily protein involved in signal transduction between hormones (Rosado et al., 2006) was up regulated at 120 hpi, and a *root meristem growth factor* 9 (RGR9), a small secreted peptide hormone (Shinohara, 2021), was down regulated between 120 and 48 hpi. Our results revealed that there is a crosstalk between the diverse hormone pathways related to soybean defense against *C. truncatum*.

### 3.7 Broad transcriptional reprogramming occurs in soybean after infection with *C. truncatum*

Selected transcription factors (TFs) and co-regulatory proteins are modulated upon pathogen recognition and signal transduction mechanisms, contributing to the activation of diverse defense mechanisms in plants (Tsuda and Somssich, 2015). In addition to the classes of TFs involved in the previously discussed signaling pathways, other 31 genes related to TF activity were modulated only in the MRP; 21 of these related to specific timings. Among these genes, NAM, ATAF CUC family (NAC), *basic helix-loop-helix protein* (bHLH), and WRKY TFs were predominant in the resistant response of soybean to *C. truncatum* (**Figure 3**).

Two homologous TFs of the NAC (ANAC002 and *Gm*NAC42) family, a bHLH and a *squamosa promoter-binding protein 1-like* (SBP1), were up regulated early, between 12 and 48 hpi, and a ANAC042 down regulated at 120 hpi. A bHLH105 was down regulated early at 48 hpi, while a bZIP (RF2b-like), a GATA14 an *ovate protein family* (OFP), *lateral organ boundaries* (LBD25), two NACs, one PLATZ, two bHLH, and two WRKY TFs had peaks of expression at 48 hpi, and were down regulated at 120 hpi. Moreover, two *growth regulation factors* (GRF4 and GRF5), two bHLHs, a pathogenesis-related gene transcriptional activator (PTI6-Like), and a PLATZ were up regulated during the necrotrophic phase, at 120 hpi (**Figure 3**).

### 3.8 Soybean defense responses are activated in the MRP upon infection with *C. truncatum*

A total of 118 genes putatively involved in several plant defense responses were specific to the MRP or the MSP; among these, 54 were modulated at specific times. For the MSP, only one gene, an aminopeptidase, was strongly down regulated (log2FC between 8.3 and 9.1) between 0 and 12 hpi. For the MRP, a *vesicle-associated protein 2-1* (PVA21) was up regulated early, between 0 and 12 hpi, and a cell wall-related protein was down regulated between 48 and 12 hpi. Later, some genes showed a peak of expression between 12 and 48 hpi, at 48 hpi, or between 48 and 120 hpi, and were subsequently down regulated. These consisted of seven cell -wall-related genes, a CHUP1 protein encoding gene, an actin 11 (ACT11), three cytochrome p450s, a DRRG R-gene, an HR -related gene, two LURP domain containing genes, an NPR1 homolog, a *constans-like* (COL13), two glycosyl transferases, three programmed cell death related, six PR-proteins, three RING-fingers, a *ripening-related protein* (SPAC24B11.05), a *random slug protein 5-like* (rsc5), five transporters, a trypsin and protease inhibitor, a U-box protein encoding genes, a VQ-motif containing protein 22 (VQ22), and a zinc finger (*constans like 16*, Col16). Curiously, ACT11 related to the actin cytoskeleton (Henty-Ridilla et al., 2013; Henty-Ridilla et al., 2014; Yu et al., 2017) is the most overexpressed gene between 12 and 48 hpi and is down regulated at the same levels at 120 hpi. Finally, a WD40 repeat-like superfamily protein, a RING finger, a remorin, a phenolic compound (F3H), two glycosyl transferases, and a *high mobility group protein 3* (HMGB3) were up regulated only at 120 hpi (**Figure 3**). The activation of putative disease-responsive genes in a coordinated response in the MRP indicates that these genes may play roles in the resistance response of soybean to *C. truncatum*. Moreover, 15 genes already reported to be involved in plant responses to abiotic stresses were modulated only in the MRP, of which six were time specific, including three with a peak of expression at 48 hpi and three up regulated only at 120 hpi (**Figure 3**).

### 3.9 Other functions revealed by the analysis that may be involved in soybean response to *C. truncatum*

Thirty-seven genes whose function in stress biotic or abiotic response is not yet characterized were DE only in the MRP and related to specific timings.

Curiously, the most overexpressed gene between 0 and 12 hpi, encoding for an alanine*–glyoxylate aminotransferase 2 homolog 3* (AGXT2), is among these. Overall, this set of genes also included allergens, genes involved in amino acid metabolism, enzymes, and genes involved in epigenetic modifications. Another five genes, without any known domain or annotation, were also DE only in the MRP at specific timings. For the MSP, only one gene related to general functions, named GEN1 endonuclease, was highly up regulated specifically at 120 hpi. The common modulation of these genes only for the MRP or MSP suggests a potential role of those in response of soybean to anthracnose.

## 4 Discussion

Considering the potential for destruction of soybean anthracnose and its constant threat to food security, the molecular basis of resistance to the disease is of great importance. Previous studies reported different levels of resistance of soybean cultivars to *C. truncatum* (Costa et al., 2009; Nagaraj et al., 2014; Yang and Hartman, 2015b), and more recently, a study investigated the molecular basis of soybean resistance to anthracnose during the reproductive stage of soybeans, in detached pods (Zhu et al., 2022). Our work focused on understanding the molecular mechanisms controlling the defense response towards different genotypes of soybean, during the early stages of the disease, which correspond to the primary inoculum of the pathogen in an area.

For this, preliminary studies aimed to identify soybean cultivars resistant to *C. truncatum*. Our results highlighted that the same soybean cultivar can present different levels of susceptibility to different strains of *C. truncatum*, and different strains of the pathogen may show different patterns. This level of interaction between plant and fungal genotypes was also observed in the following pathosystems: strawberry (*Fragaria × ananassa*)/*C. acutatum* (Simpson et al., 2006), sunflower (*Helianthus annuus* L.)/*Phoma macdonaldii* (Darvishzadeh et al., 2007), sunflower/*Sclerotinia sclerotiorum* (Davar et al., 2011), and chickpea (*Cicer arietinum*)/*Phytophthora medicaginis* (Bithell et al., 2022).

To gain a better knowledge of the molecular mechanisms involved in soybean resistance against *C. truncatum* and to explore those involved in the response of different cultivars towards different strains, we used a comparative time course transcriptomic approach. The four cultivar/strain interactions investigated showed two resistance levels: interactions 1080-*Gm1* and 1059-*Gm2* resulted in a more resistant soybean phenotype (MRP), while 1080-*Gm2* and 1059-*Gm1* interactions resulted in more susceptible ones (MSP). We compared the two combinations of the MRP with the two combinations of the MSP to investigate the differences in the plant response among both phenotypes over time. The results revealed that the transcriptomic reprogramming of the two soybean cultivars in MRP and MSP is consistent with the phenotypes observed in the four interactions, as MRPs have similar transcription patterns sharing up to 38% of DE at each time comparison, while MSPs have higher diversity of expression patterns, sharing up to 4% the DE genes within the first 12 hpi and a maximum of 0.4% in the other time comparisons (**Figure 2**). This evidence led to the hypothesis that a soybean- resistant response implies a more defined and specific reprogramming of defense genes while the MSPs do not.

**Figure 4:**
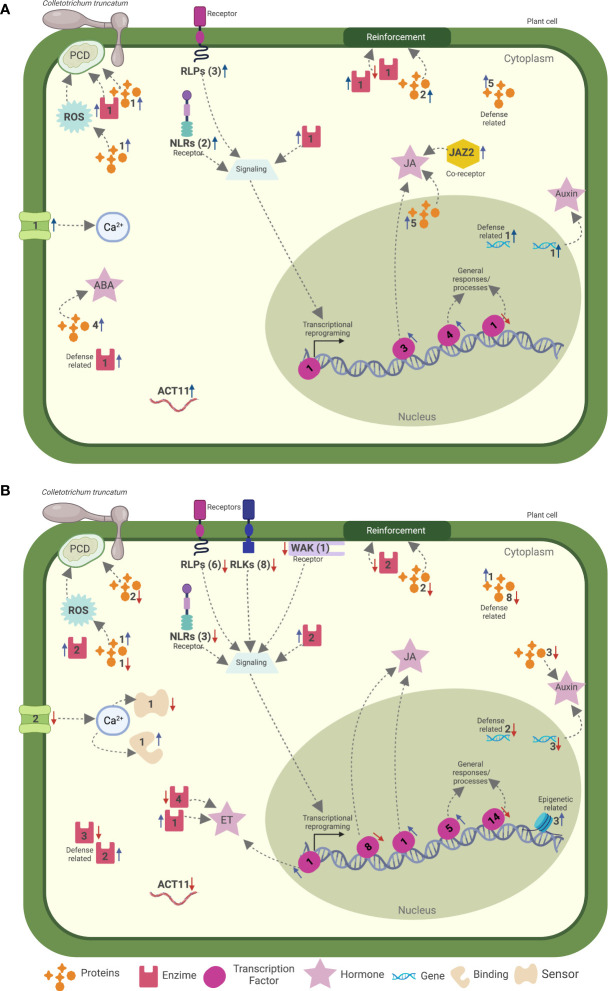
Hypothetical model for the soybean more resistant phenotype to the infection with two different strains of *Colletotrichum truncatum* during the **(A)** asymptomatic (48 vs. 12* h* post-inoculation/hpi) and **(B)** symptomatic (120 vs. 48 hpi) phases of the disease. Numbers nearby the icons are the number of genes related to the indicated process up- (blue arrows) or down regulated (red arrows). Created with BioRender.com.

The first step of an efficient plant immune system is to recognize the presence of the pathogen by PRRs and NLRs encoded by R genes at the very early stages of interaction (Jones and Dangl, 2006; Thomma et al., 2011; Cook et al., 2015; Kanyuka and Rudd, 2019). Accordingly, our results indicate that the levels of resistance of soybean upon infection with *C. truncatum* are mainly defined during the asymptomatic stage of the disease. In our study, receptors of both classes were up regulated during the asymptomatic phase of the disease in the MRP, while one Lec-RLK, an extracellular receptor, was down regulated within the first 12 hpi in the MSP. The activation of both intracellular and extracellular receptors at similar time points suggest that the recognition of *C. truncatum* by the soybean immune system relies on both layers of plant defense (**Figure 4**). Similar results have already been reported for plant defense upon infection with *Colletotrichum* spp., including strawberries inoculated with *C. fructicola*, beans (*Phaseolus vulgaris*) inoculated with *C. lindemunthianum*, and soybean pods inoculated with *C. truncatum* (Padder et al., 2016; Zhang et al., 2018; Zhu et al., 2022). Five extracellular receptors identified as differentially expressed in the MRP were also shown to be modulated early in a resistant soybean mutant after inoculation with *C. truncatum* (Zhu et al., 2022), confirming their key role in establishing a successful defense response of soybean.

The second step is represented by the signaling cascades responsible to transduce the invasion signal to the nucleus and initiate one of many defense responses involved in plant immunity (Jones and Dangl, 2006; Cook et al., 2015; Yu et al., 2017; Andersen et al., 2018). Hormone signaling was already reported to be involved in plant defense responses to *Colletotrichum* spp. (Svoboda et al., 2021). Our study revealed time-specific genes modulated in the MRP, related to Ca^2+^ fluctuations, ROS, and hormone signaling, indicating that these signaling events have a strong correlation with soybean defense mechanisms against *C. truncatum* (**Figure 4**). Similarly, the overexpression of genes involved in Ca^2+^ fluctuation and plant hormone signaling was also observed in soybean pods of a resistant mutant inoculated with *C. truncatum* (Zhu et al., 2022). An antagonistic interplay between JA and SA is suggested in the plant defense mechanisms against pathogen infection (Verma et al., 2016). Our results revealed that JA, IAA, and ABA classes of hormones responded more promptly and in a more coordinated way to *C. truncatum* infection when compared to SA, ET, and GA. Similarly, when the transcriptional profiles of detached pods of two soybean cultivars were analyzed after infection with *C. truncatum*, JA and IAA responded more strongly in the more resistant mutant, and the results were consolidated with *in vitro* tests, where spraying these classes of hormones enhanced the resistance of the more susceptible cultivar to *C. truncatum* (Zhu et al., 2022). In a previous study, when a susceptible strawberry cultivar was inoculated with *C. fructicola*, genes involved in the SA biosynthesis were up regulated while genes involved in JA biosynthesis were down regulated over time (Zhang et al., 2018). The application of JA in tea plants (*Camellia sinensis*) reduced the development of symptoms induced by *C. fructicola* (Chen et al., 2020). Altogether, this evidence suggests that JA biosynthesis and signaling are correlated with the defense responses of soybean against *C. truncatum*. Auxin signaling genes can be involved either in plant defense signaling or in increasing pathogen colonization (Denancé et al., 2013). In our work, genes related to auxin were up regulated during the asymptomatic phase of the disease, while they are down regulated during the symptomatic phase, where even more resistant combinations showed slight levels of symptom development. Similar expression patterns of auxin-related genes were observed in susceptible strawberries when inoculated with *C. fructicola* (Zhang et al., 2018).

The signal transduction that occurs after the recognition and signal perception of pathogens by the plant immune system leads to an extensive transcriptional reprogramming that is essential for the fine-tuning of plant defense (Seo et al., 2015; Tsuda and Somssich, 2015; Bian et al., 2021). In addition to the transcription factors directly involved in signaling pathways, our study revealed the time-specific modulation of 21 transcription factors after infection with *C. truncatum* (**Figure 4**), including transcripts known to be involved in plant defense mechanisms. Among the NAC superfamily, two TFs were up regulated during the asymptomatic phase of the disease, including an ANAC02, which is known to be involved in penetration resistance against biotrophic and hemibiotrophic pathogens (Jensen et al., 2007; Yuan et al., 2019), and a *Gm*NAC42, which is an essential positive regulator of the biosynthesis of the phytoalexin glyceollin in soybean that functions as a defensive metabolite against plant stresses and is involved in systemic acquired resistance (SAR) (Jahan et al., 2019). *Gm*NAC42 was already shown to be overexpressed as early as 4 hpi in soybean pods upon infection with *C. truncatum* (Zhu et al., 2022). WRKY TFs are key regulators of PTI and ETI in plants (Eulgem and Somssich, 2007). In our work, we detected seven WRKY TFs overexpressed at 48 hpi only in the MRP; two of them were time specific, namely, WRKY23 and WRKY 12. The same WRKY12 encoding gene is down regulated at 24 hpi in pods of the more resistant mutant of soybean and up regulated at the same time in the more susceptible genotype upon infection with *C. truncatum* (Zhu et al., 2022). Overexpression of WRKY23 in *Arabidopsis* enhanced its resistance to *Pseudomonas syringae* by inducing the expression of PR-1, PR-2, and PR-5 (Jing et al., 2009). Corroborating these studies, PR-2 was overexpressed only in the MRP during the asymptomatic phase of the disease and down regulated at 120 hpi, suggesting a role in plant defense against *C. truncatum*.

In plant immunity, the recognition of the plant pathogen leads to signal transduction where a transcriptional reprogramming of the plant leads to the expression of genes involved in several pathways to enhance the defense response of the plant (Jones and Dangl, 2006; Cook et al., 2015; Kanyuka and Rudd, 2019). In our work, genes known to be involved in different pathways of plant defense were activated only in the MRP. Among these, the most overexpressed gene during the asymptomatic phase of the disease was ACT11 (**Figure 4**), which is known to play an important role in PTI. The interference with actin polymerization in *Arabidopsis* increases the susceptibility to *Pseudomonas syringae* and impairs callose deposition (Henty-Ridilla et al., 2013; Henty-Ridilla et al., 2014). Moreover, the actin cytoskeleton was shown to play a central role in the non-host resistance of *Arabidopsis* to different species of *Colletotrichum* (Shimada et al., 2006) and to be related to the switch from biotrophy to necrotrophy of *C. destructivum* in susceptible *Nicotiana benthamiana* (Shan and Goodwin, 2005).

Cell wall reinforcement upon pathogen infection is considered an efficient plant defense mechanism (Huang, 2001). Several genes involved in this process, including genes related to lignin biosynthesis, namely, PME, PMEI, XTH, and KNAT2, were overexpressed during the asymptomatic phase of the disease, most of them being down regulated during the symptomatic phase (**Figure 4**). The activity of pectin methylesterases (PMEs) upon pathogen infection can favor the production of damage-associated molecular patterns (DAMPs), such as de-methyl esterified oligogalacturonides, that are recognized by WAKs to trigger plant immunity (Ferrari, 2013; Kohorn et al., 2014), while pectin methylesterase inhibitors (PMEIs) act in the post-transcriptional regulation of PMEs (Wormit and Usadel, 2018; Coculo and Lionetti, 2022). In *Arabidopsis*, mutants lacking *AtPMEI-PMEI17* showed a high susceptibility against *Botrytis cinerea* (Del Corpo et al., 2020), while AtPMEI10, AtPMEI11, and AtPMEI12 increase the resistance against the same pathogen due to pectin methylesterification of the plant cell wall (Lionetti et al., 2017). Xyloglucan endotransglucosylase/hydrolase proteins (XTH) have a potential role in plant defense against fungi (Sharmin et al., 2012). In our work, an XTH was up regulated between 12 and 48 hpi, the same gene that was overexpressed in a more resistant soybean mutant upon *C. truncatum* infection (Zhu et al., 2022).

Along with the role of ROS accumulation in HR, ROS can also be recruited by the plant immune system to create unsuitable environments for pathogen development, acting directly in signal transduction and inhibition of pathogen growth (Lamb and Dixon, 1997). Two genes encoding a *pyridoxal phosphate (PLP)-dependent transferase I* enzyme are among the highest overexpressed genes in the MRP during the early stages of *C. truncatum* infection. Little is known about the role of this enzyme in plant defense, but it has been reported in previous studies as related to oxidative stress response in strawberries upon infection with *C. acutatum* (Amil-Ruiz et al., 2016). Other members of several gene classes involved in ROS regulation were also up regulated in the MRP, including PR-15, GTPases, peroxidases, and genes involved in the phenylpropanoid pathway, *respiratory burst oxidase* (RBOH), GST, and P450. These findings suggest a role of ROS regulation during soybean defense against *C. truncatum* and go in line with other studies reporting an increase in host resistance due to the accumulation of ROS such as *Arabidopsis* when inoculated with *C. higginsianum* (Schmidt et al., 2020) and resistant genotypes of sorghum when inoculated with *C. sublineola* (Baldrich et al., 2021).

In conclusion, our study revealed a comprehensive characterization of resistant responsive genes from soybean. Our results revealed that the same soybean cultivar can activate different sets of genes upon challenge with different strains of *C. truncatum*, which can vary when the interaction results in a more susceptible or more resistant response (**Figure 4**). Interestingly, the higher resistance level of soybean against different *C. truncatum* strains resulted in several common time-specific differentially expressed genes, involved in different layers of the plant immune system, from pathogen recognition to expression of genes related to specific defense responses. Moreover, our results indicate that the asymptomatic stage of the disease is crucial for the definition of the level of susceptibility of the plant. The list of DEGs here described may pave the way to the discovery of important active host defense pathways against soybean anthracnose.

## Data availability statement

The datasets generated and analyzed for this study can be found in the GenBank under Bioproject Accession Number: PRJNA879726.

## Author contributions

TB, RB, MT, SS, and NM conceived and designed the experiments and revised the manuscript. TB and LB performed the biological experiments. TB, SB, and RB performed the bioinformatic analyses. TB and RB wrote the manuscript. TB, RB, MT, SS, LB, SB, EB, and NM contributed intellectually. All the authors read, revised, and agreed with the final version of the manuscript.

## Funding

This research was funded by The São Paulo Research Foundation (FAPESP), grant numbers 2017/09178-8 and 2021/01606-6, and by the Coordination for the Improvement of Higher Education Personnel (CAPES/PrInt), grant number 88887.368016/2019-00. This research was also supported by grants RTI2018-093611-B-I00 and PID2021-125349NB-100 from the Ministerio de Ciencia e Innovación (MCIN) of Spain AEI/10.13039/501100011033 and the European Regional Development Fund (ERDF). SB was supported by a fellowship program from the regional government of Castilla y León.

## Acknowledgments

The authors acknowledge Professor Luís Eduardo Aranha Camargo and the Molecular Genetics Laboratory of the Department of Plant Pathology and Nematology (ESALQ/USP) for technical support.

## Conflict of interest

The authors declare that the research was conducted in the absence of any commercial or financial relationships that could be construed as a potential conflict of interest.

## Publisher’s note

All claims expressed in this article are solely those of the authors and do not necessarily represent those of their affiliated organizations, or those of the publisher, the editors and the reviewers. Any product that may be evaluated in this article, or claim that may be made by its manufacturer, is not guaranteed or endorsed by the publisher.

## Supplementary material

Supplementary Figure 1
**C** truncatum specific polymerase chain reaction (PCR) results of GAPDH gene (ColF6/R5) visualized in a 3% agarose gel. M: 1 Kb leader; 1: CMES1080 positive control; CMES 1059 positive control; 3: negative reaction control; 3: Gm1 non-inoculated; 4: Gm2: non-inoculated; 6,8: 1080-Gm1; 7,9: 1059-Gm2; 10-11: 1080-Gm2.

Supplementary Figure 2 Overview of RNA sequencing data. **(A)** Number of raw reads and percentage of alignment of each library to the soybean reference genome. **(B)** Principal Component Analysis (PCA) of biological sequenced libraries in each time point. **(C)** k-means clustering analysis results and clusters that represent 75% of the genes in the More Resistant Phenotype (MRP) and the More Susceptible Phenotype (MSP). Hpi: hours post-inoculation

Supplementary Figure 3 Gene Ontology (GO) enriched molecular functions in the More Resistant Phenotype (MSP) combinations (1059-Gm2; 1080-Gm1). Up and down regulated genes at 48 vs 12 hours post inoculation (hpi) and 120 vs 48 hpi.

Supplementary Figure 4 Gene Ontology (GO) enriched biological processes in the More Resistant Phenotype (MSP) combinations (1059-Gm2; 1080-Gm1). Up and down regulated genes at 48 vs 12 hours post inoculation (hpi) and 120 vs 48 hpi.
