# Supplementary material for: Comparative transcriptomic provides novel insights into the soybean response to Colletotrichum truncatum infection
Source: Front Plant Sci. 2022 Nov 25;13:1046418. doi: 10.3389/fpls.2022.1046418 (PMC9732023; doi:10.3389/fpls.2022.1046418)
Supplement: Supplementary Figure 1 — C truncatum specific polymerase chain reaction (PCR) results of GAPDH gene (ColF6/R5) visualized in a 3% agarose gel. M: 1 Kb leader; 1: CMES1080 positive control; CMES 1059 positive control; 3: negative reaction control; 3: Gm1 non-inoculated; 4: Gm2: non-inoculated; 6,8: 1080-Gm1; 7,9: 1059-Gm2; 10-11: 1080-Gm2. [file DataSheet_1.pdf]

Supplementary Material

1. Supplementary Figures

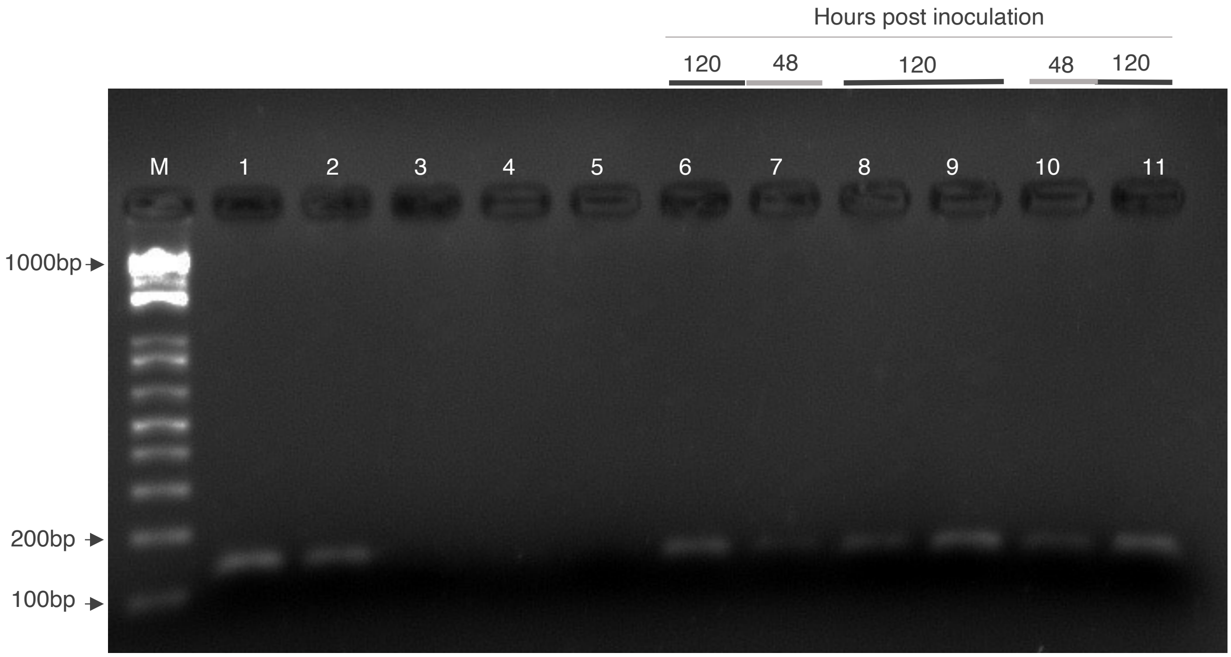

Supplementary Figure 1: *C. truncatum* specific polymerase chain reaction (PCR) results of GAPDH gene (ColF6/R5) visualized in a 3% agarose gel. M: 1 Kb leader; 1: CMES1080 positive control; CMES 1059 positive control; 3: negative reaction control; 3: *Gm1* non-inoculated; 4: *Gm2*: non-inoculated; 6,8: 1080-*Gm1*; 7,9: 1059-*Gm2*; 10-11: 1080-*Gm2*.

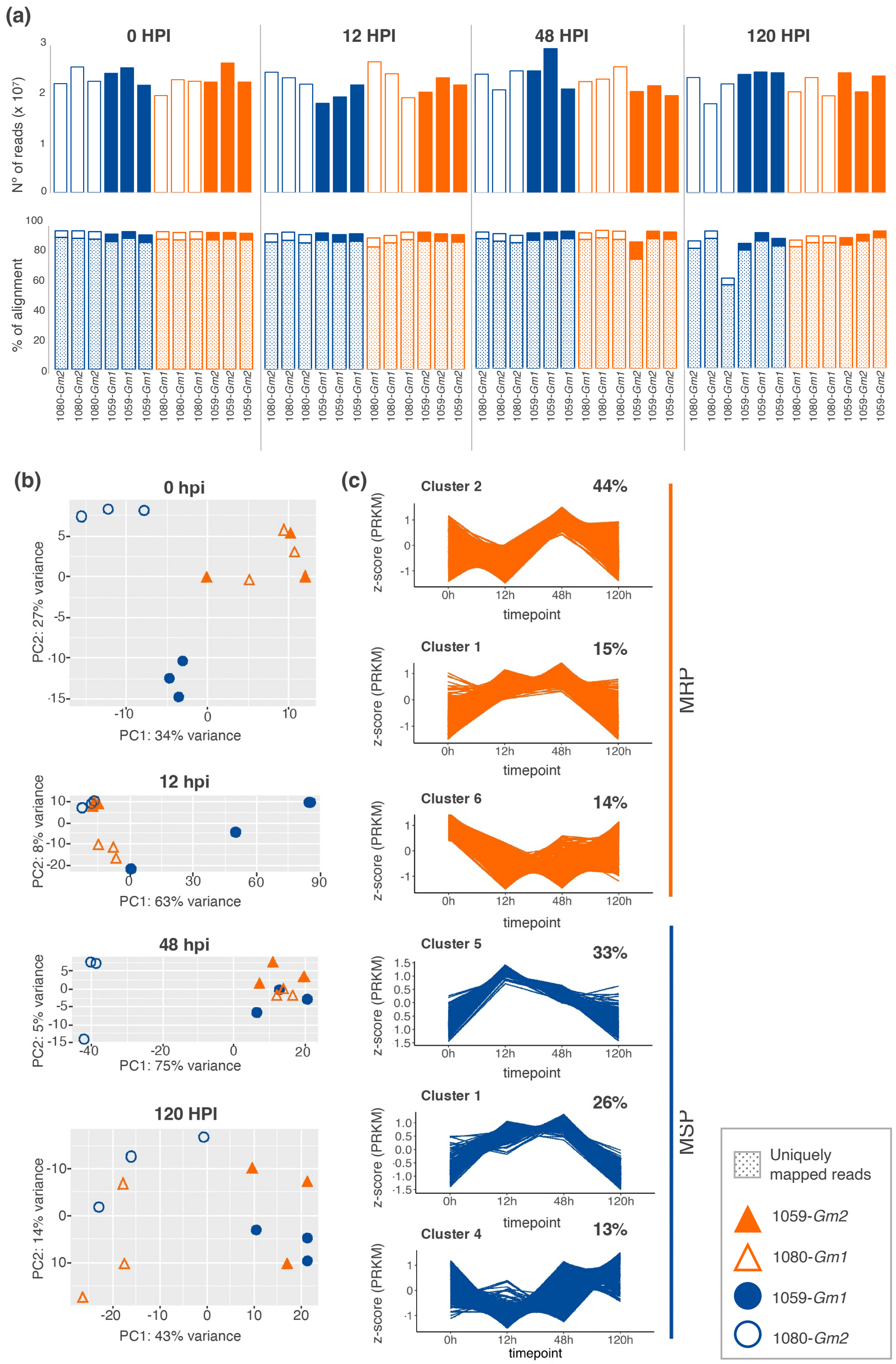

2.

Supplementary Figure 2: Overview of RNA sequencing data. (a) Number of raw reads and percentage of alignment of each library to the soybean reference genome. (b) Principal Component Analysis (PCA) of biological sequenced libraries in each time point. (c) *k*-means clustering analysis

results and clusters that represent 75% of the genes in the More Resistant Phenotype (MRP) and the More Susceptible Phenotype (MSP). Hpi: hours post-inoculation.

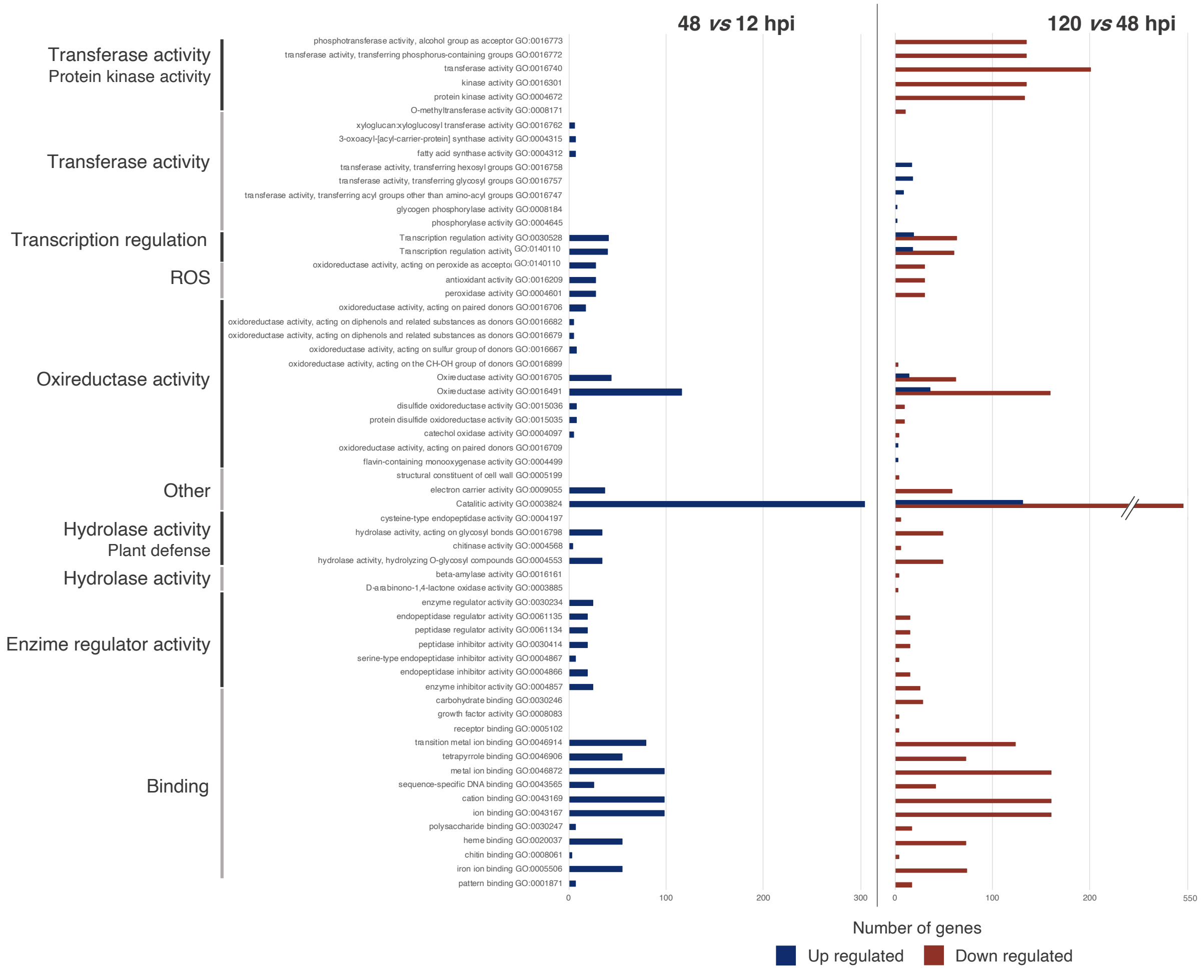

Supplementary Figure 3: Gene Ontology (GO) enriched molecular functions in the More Resistant Phenotype (MSP) combinations (1059-*Gm2*; 1080-*Gm1*). Up and down regulated genes at 48 *vs* 12 hours post inoculation (hpi) and 120 *vs* 48 hpi.

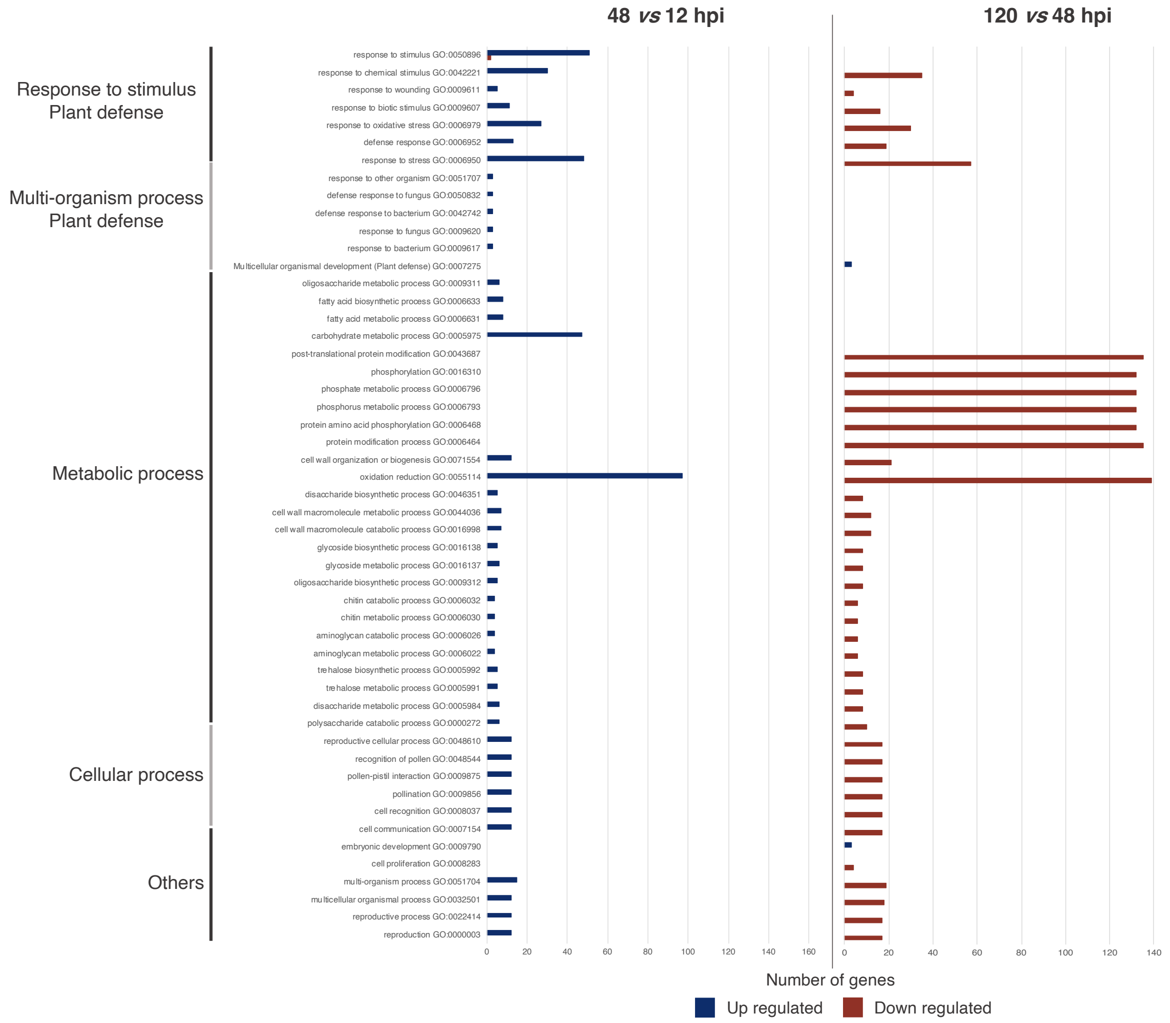

Supplementary Figure 4: Gene Ontology (GO) enriched biological processes in the More Resistant Phenotype (MSP) combinations (1059-*Gm2*; 1080-*Gm1*). Up and down regulated genes at 48 *vs* 12 hours post inoculation (hpi) and 120 *vs* 48 hpi.
